# The effect and mechanism of Internet use on the physical health of the older people—Empirical analysis based on CFPS

**DOI:** 10.3389/fpubh.2022.952858

**Published:** 2022-10-19

**Authors:** Enkai Guo, Jing Li, Le Luo, Yang Gao, Zhaohong Wang

**Affiliations:** ^1^College of P.E and Sports, Beijing Normal University, Beijing, China; ^2^College of Humanities and Law, Beijing University of Chemical Technology, Beijing, China

**Keywords:** Internet use, the older people, physical health, physical exercise, mediation effect

## Abstract

The use of the Internet has a promoting effect on the physical health of the older people. However, previous studies are mostly focused on the perspective of the overall population, or limited to the direct effects, ignoring the exploration of the mechanism of action and the perspective of the older people. Based on the data of the China Family Panel Survey (CFPS) in 2014, 2016, 2018, and 2020, this study found that the use of the Internet has a significant effect on the physical health of the older people, especially among the population groups of females, rural residents, and those living in central and western regions of China. In addition, this study also found that the use of the Internet by the older people can increase their exercise frequency, thereby improving their physical health. Therefore, to promote active aging, this study proposes to further increase the popularity of the Internet among the older people, encourage the introduction of age-appropriate Internet systems and sports facilities, create an online fitness platform for the older people, and promote scientific fitness programs for them.

## Introduction

Since the 21st century, the growth of the aging population has become an increasingly prominent challenge in countries around the world, and it is expected that the population aged 60 and above will reach 25% by the middle of this century (1). The WHO pointed out that the global population aged 60 and over was 1 billion in 2019, and will grow to 2.1 billion by 2050, where a more significant increase would be found in developing countries (2). Taking China as an example, as of November 2021, its population aged 60 and above reached 264 million, accounting for 18.70% of the total volume, an increase of 5.44% over 2010. Among them, the number of people aged 65 and above reached 191 million, an increase of 4.63% over 2010 (3). With the increase of age, the immune function of the human body weakens, and the prevalence of chronic diseases, comorbidities, and other basic diseases gradually increases. During the COVID-19 epidemic, most critically ill patients are older people, and their susceptibility to diseases may often lead to increasingly severe health conditions. In less-developed areas, a shortage of advanced medical service facilities, national fitness venues, and social security measures jeopardizes the effort to improve the physical health of the older people. Therefore, the Chinese government has continuously increased its emphasis on the health of the older people and has issued relevant policies to promote the health care consumption of the older people (4).

Simultaneous with the aging process of the population is the rapid development of the Internet industry. So far, the popularity of the Internet in countries around the world is on the rise. Many studies have found that the number of “silver surfers” is increasing in countries and regions such as the United States and Europe due to the enormous benefits of the Internet for aging populations (5). For example, the Internet can help the older people strengthen their social connections while increasing their offline leisure and recreational activities at the same time (6). Therefore, many countries advocated the construction of age-appropriate venues, facilities, and equipment using sports intelligence and Internet technology to facilitate the older people's participation in sports, hence helping them to be healthy and achieve active aging (7). It can be seen that the Internet is closely related to the daily life of the older people. It is not only a guarantee to meet their basic living needs in the information age, but also an important way to promote their physical health and improve their quality of life.

Therefore, this study focuses on the older people and uses the micro-panel data of China Family Panel Studies (CFPS) in 2014, 2016, 2018, and 2020 to analyze the impact of Internet use on the physical health of the older people. This study aims to: (1) explore whether there is a relationship between Internet use and the physical health of the older people; (2) if so, determine the direction of the relationship; (3) verify the relationship between exercise behavior and the physical health of the older people.

## Literature review

The continuous advancement of the “Internet +” action has brought a huge impact on the lives of residents. In an aging society, the increased use of the Internet has provided technical and informative sources for the older people to maintain a healthy physical state. The WHO classifies people aged 60 and above as the older people (8). Most studies show that the most frequent use of the Internet in the older people group is among those between the ages of 60 and 70 (9, 10). In addition, factors such as occupation, education, and family economic status before retirement will also affect the frequency of using the Internet (11). In the existing research, few scholars directly correlate Internet use with the physical health of the older people in China. Usually, the physical health of the older people is used as a mediator or moderator to study the impact of Internet use on mental health (11), social participation (12), and subjective wellbeing of the older people (13). In related research, domestic and foreign scholars mostly study the impact of Internet use on the health of the older people from the perspectives of sociology and psychology. Consequently, two major views have been formed, namely the “network gain effect theory”, which believes that Internet use has a positive impact on the health of the older people; and the “presence substitution effect theory”, which believes that Internet use has no significant relationship with the health of the older people, and may even have a negative impact (14).

First, in the “network gain effect theory”, some scholars found that older people may acquire knowledge of disease prevention and health care through online publications *via* channels such as WeChat, which is helpful to improve their health level (15, 16). Jiang and Chen (17) used the physical and mental health of the older people as a mediating variable and proved its positive mediating utility in the impact of Internet use on the subjective wellbeing of the older people. Moreover, it has also been confirmed that the health level of the older people who use the Internet to obtain health information is significantly higher than those who do not use the Internet (17). Lv et al. (18) found that the use of the Internet can also improve the physical health of the older people, thereby promoting their employment and social participation (18). Thus, the Internet is a conducive tool to fully leverage the human capital of the older people, relieving the social pension pressure.

Second, in the “presence substitution effect theory”, some scholars pointed out that Internet technology will occupy the social and outdoor sports duration of the older people, narrow the social network of the older people, and form bad living habits, which is not conducive to the physical health of the older people (19). He and Yan used the Probit model to analyze the data of the China Longitudinal Aging Social Survey (CLASS) and found that the Internet has isolated the older people from the community, replacing the offline participation of the older people in community sports and entertainment activities (20). Taken together, the two effect theories correspond to the two development paths of an aging society in the Internet era. Based on this, this study proposes a set of competing hypotheses:

H1a: The physical health level of the older people who use the Internet is significantly higher than those who do not use the Internet.

H1b: The physical health level of the older people who use the Internet is significantly lower than those who do not use the Internet.

In fact, there are still prominent problems such as urban-rural differences and unbalanced regional development in China, which may lead to a certain degree of heterogeneity in the use of the Internet by the older people. In addition, the social characteristics of the older people themselves also have a certain degree of heterogeneity. For example, because women are more accustomed to using the Internet for shopping or socializing, the Internet has a significantly higher inhibitory effect on rural women's sports participation behavior than men, and has a more significant inhibitory effect on women's physical health (21). Differences in areas such as urban-rural gap, physical conditions, incomes, and social status also cause the divergence in data gathered among the older people (22). Therefore, this study proposes the second research hypothesis:

H2: The use of the Internet has different effects on the physical health of the older people living in different regions, with different household registrations and genders.

Existing research shows that whether it is through social networking, media publicity, popular science APPs, or traditional websites, the information acquired will have an impact on the old people's participation in physical exercise (15). The main mechanism is that the older people can obtain medical, health care, and other related knowledge from different channels through the use of the Internet. Therefore, when they are not sick or suffering from chronic diseases, they may participate more in physical activities in the community, squares, and so on for their physical health. Wang et al. used the data from the Chinese General Social Survey (CGSS) in 2017 and used a logistic model to find that Internet use significantly promoted both the frequency and intensity of exercise among the older people (23). Relevant foreign studies also pointed out that the older people who use the Internet demonstrate a higher frequency of active exercise. In poverty-stricken areas, this conclusion is even more obvious, and the inequality between the two group is even more severe as the older people who do not use the Internet have a stronger sense of loneliness and a higher degree of social isolation (24). Therefore, based on Hypothesis 1, to further explore the impact mechanism of Internet use on the physical health of the older people, this study introduces the mediating variable of physical exercise to study its mediating mechanism, and proposes the third research hypothesis:

H3: The use of the Internet can promote physical activity in the older people, which in turn affects their physical health.

Although this study suggests that physical activity may be a mediator of the effects of Internet use on the physical health of the older people, we believe that such a factor may not be sufficient to explain all the differences in the effects. That is, in addition to physical exercise, there may be other mediating variables at the same time. Therefore, this study only attempts to explore the role of physical exercise as an intermediary variable from an exploratory perspective.

## Materials and methods

### Data sources

The research data in this paper comes from the China Family Panel Studies (CFPS) database (Data access online: http://www.isss.pku.edu.cn/cfps/). CFPS collects data from three dimensions: individual, family, and community, and adopts various forms of questionnaires such as long, short, pick-up, and telephone interviews, aiming to reflect the development and changes in China's society, economy, population, health and education. To maintain the period of the data and the consistency of variables, this study used the survey data of 2014, 2016, 2018, and 2020 with high similarity in the questionnaires. In this study, the samples aged 60 and above were screened, and after excluding the samples with severe missing variables, the total number of samples included in this analysis was 32,947, where the numbers of valid samples in 2014, 2016, 2018 and 2020 are 8,414, 9,113, 9,251, and 6,169, respectively. All results in this study were calculated by Stata software.

### Variable selection

#### Dependent variable

This study leveraged the method of Du and Wang by using self-rated health status to measure health level (14), and the selected dependent variable is the self-rated health status of the older people. The question in the questionnaire is “How do you think your health is?”, the answer to the question ranges from “very healthy” to “very unhealthy”, at the rate of 1–5. As shown in Table 1, the average self-assessed physical health of the older people is 3.587, indicating that more older people are in a “relatively healthy” state. In 2014, 2016, 2018, and 2020, the self-assessed physical health status of the older people was 3.60, 3.65, 3.59, and 3.49, respectively, showing that the physical health status of the older people is getting better.

**Table 1:** Descriptive statistical results of key variables.

**Categories**	**Variables**	**Coding values**	**Mean**	**SD**
Dependent variable	Self-rated health status	Measured on a scale of 1 (very healthy) to 5 (very unhealthy)	3.587	1.216
Independent variable	Internet use	Use = 1, not use = 0	0.101	0.301
Mediating variable	Exercise frequency		3.728	3.689
**Controlled variables**				
Social characteristics	Gender	Male = 1, female = 0	0.488	0.500
	Age	Measured in years	69.341	7.395
	Marital status	Married = 1, unmarried = 0	0.987	0.112
	Education	Measured in years	4.566	4.510
	Family count	The number of family members living together	2.842	3.971
	Political status	Communists = 1, non- Communists = 0	0.004	0.345
	Subjective social status	Measured on a scale of 1 (low) to 5 (high)	3.231	1.134
Economic characteristics	Family medical expenditure	Take logarithm	7.869	1.452
	Subjective economic income status	Measured on a scale of 1 (low) to 5 (high)	2.551	1.428
	Subjective wellbeing	Measured on a scale of 0 (low) to 10 (high)	3.194	7.315
Regional characteristics	Urban	Urban = 1, rural = 0	0.472	0.499
	Economic area	Eastern region = 1, Central region = 2, North-eastern region = 3, Western region = 4	2.340	1.205

#### Independent variable

**Figure 1:**
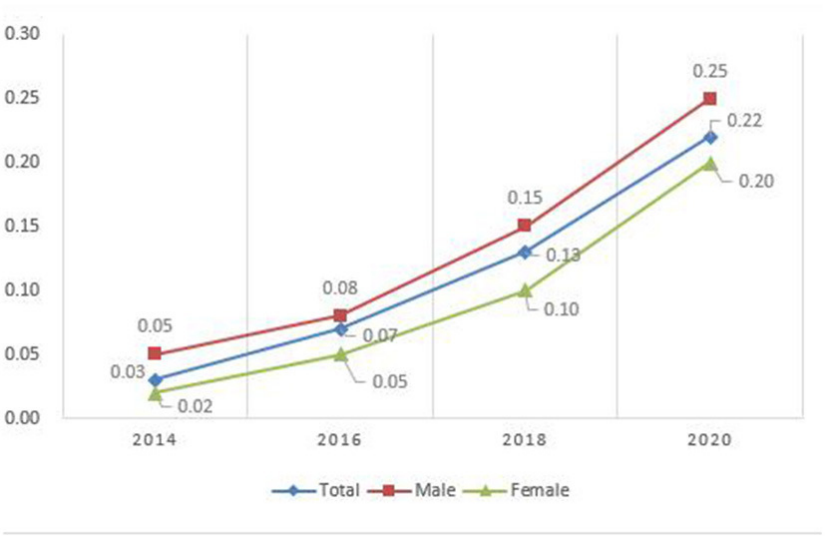
Changing trend of Internet usage frequency among the elderly.

The core independent variable of this study is whether to use the Internet, including mobile Internet access and computer Internet access. The descriptive statistics in Table 1 show that the proportion of the older people who use the Internet is relatively low, only 10.1%. Figure 1 shows that the Internet usage rate of the older people has increased significantly in recent years, an increase of 19%, and the increase in males is even greater, from 0.05 to 0.25, an increase of 20%. In the robustness test, this study selects four variables: spare time online, the importance of the Internet, the importance of using the Internet to learn, and the importance of using the Internet to socialize as independent variables to verify the robustness of the model in this study.

#### Controlled variables

This study refers to the existing literature and selects control variables from three aspects: social, economic, and regional. Among them, the personal social characteristics include gender, age, marital status, education, political status, and subjective social status, a total of seven variables. Of all the samples enrolled, female older people account for 48.77%, which is slightly lower than that of male older people; the average age of the older people is about 69 years old, and the majority of the relatively younger ones in the group are between 60 and 70 years old; the average number of years of education for the older people is about 5 years; The number of Communist Party members is significantly lower than that of non-party members. The economic characteristics mainly include family medical expenditure, individual subjective economic income status and subjective wellbeing. The data shows that most of the older people enrolled believe that their economic income is among average level, and the proportion of those who scored 3 is 37.66%, which is much higher than other scores. The regional characteristics mainly include two variables: urban-rural residence and economic regions. In the 2014–2020 survey, the ratios of older people living in urban areas were 46.58, 47.12, 47.28, and 48.16%, respectively, showing that the number of older people in rural areas is still relatively large, while the number of their peers in urban areas is under significant growth. Meanwhile, according to the division of China's economic zones by the National Bureau of Statistics of China, this study divides the regions into four regions: eastern, central, north-eastern, and western (25). Among them, the eastern region has the largest sample size of older people, accounting for 34.47%, while the northeast region accounts for the least, only 14.60%.

In terms of mediating variables, a large number of studies have pointed out that residents can access and master sports-related knowledge and publicity content through the Internet, thereby stimulating their motivation to participate in sports, that is, the use of the Internet has a significant impact on residents' participation in physical exercise. Therefore, this study refers to the method of Wang et al. (23), and uses the exercise frequency of residents to measure their enthusiasm for physical exercise, and uses it as a mediating variable to explore the impact mechanism of Internet use on the physical health of the older people.

### Model construction

In order to examine the impact of Internet use on the health of the older people and its heterogeneity, according to the characteristics of the dependent variable, that is, ordered categorical variables, the ordered logistic regression model (ordered logit) was used to construct the benchmark regression model of this study:


(1) $$Y_{i}=\beta_{0}+\beta_{1}X_{i}+\sum \beta_{k}Z_{k,i}+\varepsilon_{i}$$

Where, Y_i_ is the explained variable, which represents the subjective health status of the i-th older person. X is an explanatory variable, indicating whether the i-th older person uses the Internet. Z_k_ (k = 1…11) is a control variable, including gender, age, marital status, years of education, number of family members, political status, subjective social status, family medical expenditure, subjective economic income status, etc. ε is a random disturbance term, which obeys the Logistic distribution. β_0_ is the intercept term, and β_1_ and β_k_ are parameters to be estimated.

## Results

### Basic regression

Table 2 is based on the 4-year survey data from 2014 to 2020, showing the regression results of whether the use of the Internet affects the physical health of the older people. Column (1) mainly examines the influence of each controlled variable on the dependent variable (self-rated health status). Column (2) introduces the independent variable (Internet use) on the basis of the previous step. Column (3) is the overall regression result after including the mediator variable (exercise frequency). From (1) to (3), there is a slight increase in Pseudo *R*^2^, indicating a certain improvement in the overall fit of the model.

**Table 2:** Baseline regression results.

**Variables**	**(1)**	**(2)**	**(3)**
Internet use		−0.123*** (0.040)	−0.118*** (0.040)
Exercise frequency			−0.027*** (0.004)
Gender	−0.341*** (0.026)	−0.342*** (0.026)	−0.341*** (0.026)
Age	0.013*** (0.002)	0.012*** (0.002)	0.013*** (0.002)
Marital status	−0.229* (0.117)	−0.231** (0.117)	−0.232** (0.117)
Education	−0.023*** (0.003)	−0.021*** (0.003)	−0.018*** (0.003)
Family count	−0.040*** (0.006)	−0.041*** (0.006)	−0.043*** (0.006)
Political status	−0.073** (0.032)	−0.077** (0.032)	−0.064** (0.032)
Subjective social status	−0.035*** (0.013)	−0.036*** (0.013)	−0.034*** (0.013)
Family medical expenditure	0.275*** (0.009)	0.276*** (0.009)	0.277*** (0.009)
Subjective economic income status	−0.130*** (0.010)	−0.130*** (0.010)	−0.128*** (0.010)
Subjective wellbeing	−0.153*** (0.007)	−0.152*** (0.007)	−0.149*** (0.007)
Urban	−0.168*** (0.026)	−0.159*** (0.026)	−0.138*** (0.026)
Year	Controlled	Controlled	Controlled
Economic area	Controlled	Controlled	Controlled
Pseudo *R*^2^	0.037	0.037	0.038
*N*	22,591	22,591	22,591

*p < 0.1, **p < 0.05, ***p < 0.01.

Robust standard error for *t*-statistics in parentheses.

The results in column (1) show that the estimates of the controlled variables are as expected. In terms of personal social characteristics, the physical health level of male older people is higher than that of female older people. In terms of age, there is a significant negative correlation between the age of the older people and their physical health, that is, with the increase of age, their physical health decrease significantly. Older adults with more years of education are healthier than those with less education. Older people with more family members have higher levels of physical fitness than those with relatively few family members. In addition, economic characteristics also showed that at the 1% level, the subjective social and economic status of the older people also had a significant positive correlation with their physical health. In terms of regional characteristics, the physical health of the urban older people are better than that of the rural older people.

According to the characteristic that the dependent variable is ordinal, this study uses the logit model for estimation. In column (2) of Table 2, it is found that Internet use is significantly positively correlated with the physical health of the older people, and it is significant at the 1% level when the controlled variables are controlled, which validates H1a and rejects H1b. Column (3) shows that after the introduction of exercise frequency, there is also a significant positive correlation between physical exercise and the level of physical fitness of the older people. That is, the higher the frequency of exercise, the higher the level of physical fitness of the older people.

### Robustness test

Although the variable “Internet use” is an intuitive indicator to measure the Internet access of the older people through mobile or computer, it cannot reflect the degree of Internet use by the older people due to the nature of the dichotomous variable. For example, it is difficult to measure whether the Internet is used singularly or frequently, or the main purpose of using the Internet. To this end, this study uses questions related to “how important is obtaining information when using the Internet”, “how important is learning when using the Internet”, and “how important is social interaction when using the Internet” to replace independent variables. The results in Table 3 show that after replacing the independent variables, the regression results are basically consistent with the benchmark regression. That is to say, the higher the older people emphasize the importance of the Internet and its use in learning or socializing, the more likely they are to have better physical conditions. In conclusion, the above analysis results further show that the model estimation structure of this study has good robustness.

**Table 3:** Robustness test results.

**Variables**	**(1)**	**(2)**	**(3)**
Importance of internet	−0.061*** (0.012)		
Importance of internet to learn		−0.014*** (0.004)	
Importance of internet to socialize			−0.013*** (0.004)
Controlled variables	Controlled	Controlled	Controlled
Year/economic area	Controlled	Controlled	Controlled
Pseudo *R*^2^	0.037	0.037	0.037
*N*	22,591	22,591	22,591

***p < 0.01.

Robust standard error for t-statistics in parentheses.

### Endogenous test

To eliminate the selection bias of the research sample, this study used the Heckman model for endogeneity treatment. The endogeneity test results are shown in Table 4. The λ in the second-stage OLS model is 0.230, which is significant at the 1% level. It shows the rationality of the Heckman model and that there is indeed a selection bias in the sample of this study. After eliminating the selection bias and addressing the endogeneity problem caused by missing variables, the use of the Internet by the older people still has a significant positive effect on their physical health, which is consistent with the previous verification results. H1a is hence verified again.

**Table 4:** Test results of Heckman model.

**Variables**	**First stage (Probit)**	**Second stage (OLS)**
Internet use		−0.496*** (0.060)
Λ		0.230*** (0.035)
_cons	0.282 (0.218)	3.644*** (0.010)
Controlled variables	Controlled	Controlled
Pseudo *R*^2^/rho	0.250	0.191
*N*	22,593	22,593

***p < 0.01.

Robust standard error for t-statistics in parentheses.

### Heterogeneity analysis

It can be seen from the foregoing that the influence of Internet use on the physical health of the older people is heterogeneous in terms of gender, urban, and economic area. Therefore, this study conducts heterogeneity analysis from these three aspects. As shown in Table 5, the impact of Internet use on female older people, rural older people, and the older people in the central and western regions is more significant, and all of them are significant at the 5% level. Thus, H2 is partially verified.

**Table 5:** Heterogeneity test results.

**Variables**	**Gender**		**Urban**		**Economic area**			
	**(1) Male**	**(2) Female**	**(3) Urban**	**(4) Rural**	**(5) Eastern**	**(6) Central**	**(7) South-eastern**	**(8) Western**
Internet use	−0.054 (0.053)	−0.222*** (0.062)	−0.084* (0.050)	−0.250*** (0.081)	−0.040 (0.063)	−0.272*** (0.081)	−0.053 (0.097)	−0.234** (0.097)
Controlled variables	Controlled	Controlled	Controlled	Controlled	Controlled	Controlled	Controlled	Controlled
Year	Controlled	Controlled	Controlled	Controlled	Controlled	Controlled	Controlled	Controlled
Pseudo *R*^2^	0.036	0.031	0.034	0.039	0.038	0.037	0.046	0.034
*N*	11,297	11,294	10,628	11,963	7,925	5,569	3,326	5,779

*p < 0.1, **p < 0.05, ***p < 0.01.

Robust standard error for t-statistics in parentheses.

### The mechanism of internet use on the physical health of the older people

Currently, the mechanism of action between Internet use and the physical health of older adults is unclear. However, physical activity is often considered an important foundation for improving physical health. Therefore, to further explore the issue, this study selected exercise frequency as a mediating variable to verify its mediating mechanism. On the basis of previous research, this study refers to the research ideas of Wang et al. (23), and uses structural equation modeling to verify the mediating effect. Table 6 presents the results of the mediating effect of the frequency of physical exercise among the older people on the impact of Internet use on physical health. The results show that Hypothesis 3 holds.

**Table 6:** Results of the mediating effect of exercise frequency on the influence of Internet use on the physical health of the elderly.

	**β**	**SE**	**95% CI**	***P*-value**
Total effect	−0.219	0.024	(−0.262, −0.175)	0.000
Direct effect	−0.171	0.025	(−0.218, −0.128)	0.000
Indirect effect	−0.048	0.004	(−0.058, −0.040)	0.000

Bootstrap sampling times is 500 times.

**Figure 2:**
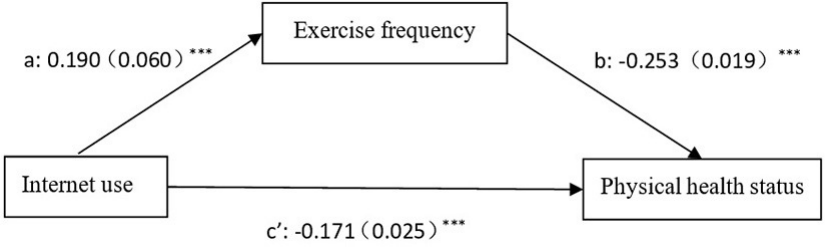
Path diagram of the influence of Internet use and exercise frequency on the physical health of the elderly.

Figure 2 more clearly shows the positive mediating role of exercise frequency in the process of Internet use affecting the physical health of the older people. The results showed a significant positive correlation between Internet use and exercise frequency. Internet use and exercise frequency were also significantly positively correlated with the physical health status of the older people. The indirect effect of exercise frequency on the physical fitness of the older people was significant at the 1% level, and the effect was about 25%. These results suggest that exercise frequency has a significant mediating effect between Internet use and physical health in the older people, with a mediating effect of 0.048 (a^*^b), which is significant at the 1% level.

## Discussion

### Summary

In this study, we used 4 years of CFPS data to examine the impact of Internet use on physical health in older adults by assessing possible heterogeneity and mediating mechanisms. The results show that, under the premise of including control variables, Internet use has a significant effect on the physical health of the older people, which is consistent with some existing research results (11, 22, 23, 26). In terms of controlled variables, the physical health of the older people decreased significantly. This is consistent with the law of human growth (27), because aging is often accompanied by the degradation of bodily functions, such as calcium loss, hearing and visual decline, decreased response ability, and decreased immune resistance of the body. The longer the years of education, the better the health of the older people, and the “happiness effect” of education is gradually highlighted. This may result from the role education plays in boosting people's awareness of scientific exercise and regular lifestyle, and providing theoretical know-how for the improvement of physical health (28). In addition, empty nesters tend to have worse health than those with children and grandchildren around their knees. With the increase in the number of relatives around, the social time and frequency of the older people have increased significantly, and the rich daily life allows the older people to experience the warmth and need of the family, which in turn helps to improve physical and mental health (29). Moreover, social status and economic income are also important factors affecting the health of the older people. The older people with higher economic income and social status have sufficient material security to support their healthy life in their leisure time, such as regular participation in outdoor sports activities and community events (30).

The results of heterogeneity analysis show that the impact of Internet use on the health of the older people varies between genders, urban-rural areas, and economic zones. The health promotion effect of using the Internet on female older people is significantly higher than their male counterparts. The possible reason is that the older people enrolled in this study are a generation who grew up when the whole society had a prevailing “preference for sons”, giving the female group a stronger craving for social resources (31). In the new era, this craving has evolved into the acceptance of new things, that is, female older people are more willing to try new things to prove that they have an equal position in social resources. Therefore, in the Internet age, female older people are more willing to obtain information through the Internet, such as health information, shopping information, social information, etc. Under the influence of the national strategy of “Healthy China”, the older women's awareness of their health has increased significantly, and their physical health conditions have been effectively improved by actively collecting health-related information. In terms of urban-rural differences, the use of the Internet has a more significant effect on physical health promotion of the rural older people. This may largely result from the dual structure in China where a significant gap can be found in many facets between urban and rural areas for a long time, such as government financial expenditure, Internet penetration level, and infrastructure construction. Boosting the rural older people's access to the benefits of scientific achievements is an important task in China's poverty alleviation campaign (32). The sample group in this study comes from 2014 to 2020, which coincides with the year in which poverty alleviation was carried out. It shows that with the effort of the government to bring technology into the countryside, the health level of the older people in rural areas has been fully improved due to the influence of the Internet. As for economic zones, the effect of Internet use on the physical health of the older people in the central and western regions is more significant. This may be due to differences in the level of economic development, environmental quality, and urban infrastructure in different regions. As a result, older people living in areas where technologies such as the Internet have developed late have a higher interest in technology and higher expectations (33). Generally speaking, the healthy aging of the older people in the eastern region is better, while the sub-health status of the older people in the central and western regions is more common (34). Therefore, with the spread of the Internet, although the physical health level of the older people in the eastern and north-eastern regions has improved, the improvement rate is relatively low due to their higher overall health level. On the contrary, under the influence of the Internet, the overall health of the older people in central and western regions has been greatly improved through scientific information and methods.

The results of mediation analysis showed that Internet use was positively correlated with physical exercise behavior in the older people, which was consistent with previous research results (21, 35). The Internet helps to broaden the channels for the older people to acquire health knowledge and protect their fitness rights and interests (36). Therefore, the older people can use the Internet to grasp relevant information such as national fitness and scientific fitness. With the continuous participation of the older people in physical activities to strengthen their bodies, whether it is square dance, Tai Chi, jogging, or other forms of aerobic exercise, the physical function of the older people has been improved and physical discomfort has been alleviated (37). This helps to cultivate the fitness awareness of the older people, promote them to become a sports population, and then improve the physical health of the older people.

The difference in the frequency of the older people's participation in outdoor sports will inevitably affect their health. For example, the health benefits of regular outdoor sports for a month are significantly higher than that of a single day, and residents who seldom or never participate in outdoor sports within a year generally have worse physical health (38). This may be because long-term regular exercise is beneficial for the older people to recover after appropriate exercise, and this knowledge can usually be found on the Internet. In general, the physical recovery level of the older people is significantly lower than that of the young. Therefore, the older people who do outdoor exercise every day may not be able to recover the physical function level, which greatly reduces the exercise effect and even has an adverse effect on their health. However, older people who are less active and do not participate in outdoor sports are unable to enjoy the benefits of exercise. This may be because they have not yet learned the benefits of exercise from the Internet, so they lack motivation to help them change their existing habits.

### Policy Implication

The conclusions drawn from this study have several important policy implications. First, speed up the popularization of the Internet among the older people in China. According to relevant data, although there are nearly 1 billion Internet users in China, the number of older people using the Internet is still relatively small (21). Therefore, in the context of “Internet +”, the infrastructure of the Internet should be strengthened, and the accessibility of the Internet for the older people should be enhanced through publicity lectures, community volunteer services, and family counseling. This measure will help the older people to master the use methods and operating procedures of the Internet, computers, smartphones, etc., and prevent the older people from being isolated by the information society.

Second, encourage the introduction of age-appropriate Internet systems and sports facilities. With the development of an aging society, it is urgent to meet the older people's need for the Internet and sports & fitness from the supply side. On the one hand, on the basis of meeting the needs of the older people to obtain information, socialize, and study, it will simplify the Internet usage process, and launch a special model for the older people in a timely manner to optimize the Internet experience of the older people. On the other hand, a special area for sports for the older people is divided. According to the sports needs of the older people in the region, introduce older people-friendly fitness facilities and fitness equipment, and increase the participation of the older people in physical exercise through simple, convenient and high-safety sports facilities.

Third, through platforms such as TikTok, create an online fitness platform for the older people and promote scientific fitness programs for them. Encourage communities and gyms to work with professional nutritionists to formulate targeted scientific fitness and nutrition programs for the older people with different regions, genders, ages and other characteristics. And build an online fitness platform for the older people through the Internet to provide the older people with convenient, professional and comprehensive exercise guidance, thereby improving the physical health of the older people.

### Strengths

This study has several advantages. First, it examines the mediating role of exercise frequency in the relationship between Internet use and physical fitness in older adults. This not only complements previous research, but provides new evidence for understanding the health status of older adults and the benefits of physical activity. Second, this study uses CFPS data across years to construct panel data to estimate the impact of Internet use on the physical health of older adults. This helps to reflect more comprehensively the physical health of the older people and the changing attitudes toward the Internet. Third, this study uses the Heckman two-stage model, which eliminates the endogeneity problem caused by sample selection bias and ensures the robustness of model estimation.

### Limitations

There are still some shortcomings in this study. First, this study only measures the physical health level of the older people from the subjective level, and lacks a description of the objective health level. In the future, we will continue to collect empirical data to further explore the impact of Internet use on the objective physical fitness of the older people. Second, this study only measured the exercise behavior of the older people from in terms of exercise frequency, and lacked the investigation of exercise duration and exercise motivation. In the future, we will continue to expand the research indicators related to exercise behavior for further exploration and interpretation.

## Conclusion

With the further development of informatization and aging, the Internet has gradually become an important channel for the older people to obtain information. This study found that Internet use has a promotion effect on the physical health of the older people. Moreover, exercise frequency is an important channel linking Internet use and physical health of the older people, and has a partial mediating effect between the two. Therefore, to support people to age well, we should speed up the popularization of the Internet among the older people, and introduce fitness facilities suitable for aging, so as to provide guarantees for the improvement of the health of the older people.

## Data availability statement

The datasets presented in this study are publicly available via the China Family Panel Studies (CFPS) database. Access data online *via* http://www.isss.pku.edu.cn/cfps/. Further queries can be directed to the authors.

## Ethics statement

The studies involving human participants were reviewed and approved by Peking University Biomedical Ethics Committee. The patients/participants provided their written informed consent to participate in this study.

## Author contributions

EG and ZW: theme. EG: methodology. EG and LL: software. YG: resources. EG and JL: original draft. JL, LL, and ZW: review and editing. ZW: supervision and funding acquisition. All authors have read and agreed to the published version of the manuscript.

## Funding

This research was funded by the National Social Fund of China, Beijing, China, Grant number: 22ATY003; Decision Consulting research project of General Administration of Sport of China in 2019, Beijing, China, grant number: 2019-C-09.

## Conflict of interest

The authors declare that the research was conducted in the absence of any commercial or financial relationships that could be construed as a potential conflict of interest.

## Publisher's note

All claims expressed in this article are solely those of the authors and do not necessarily represent those of their affiliated organizations, or those of the publisher, the editors and the reviewers. Any product that may be evaluated in this article, or claim that may be made by its manufacturer, is not guaranteed or endorsed by the publisher.

## References

[1] United Nations. World Population Ageing Report. Available online at: https://www.un.org/en/development/desa/population/publications/pdf/ageing/WPA2015_Report.pdf (accessed August 6, 2022).

[2] World Health Organization. Ageing. Available online at: https://www.who.int/health-topics/ageing#tab=tab_1 (accessed August 31, 2022).

[3] National Bureau of Statistics. Main Data of the Seventh National Population Census. Available online at: http://www.stats.gov.cn/tjsj/zxfb/202105/t20210510_1817176.html (accessed May 11, 2021).

[4] Guo E Zhong H Gao Y Li J Wang Z. Socioeconomic disparities in health care consumption: using the 2018-China family panel studies. Int J Environ Res Public Health. (2022) 19:7359. 10.3390/ijerph19127359 35742607 PMC9224432

[5] Gilleard C Higgs P. Internet use and the digital divide in the English longitudinal study of ageing. Eur J Ageing. (2008) 5:233–9. 10.1007/s10433-008-0083-7 28798575 PMC5546327

[6] Quittschalle J Stein J Luppa M Pabst A Löbner M Koenig H. Internet use in old age: results of a German population-representative survey. J Med Internet Res. (2020) 22:e15543. 10.2196/15543 33226351 PMC7685698

[7] The General Office of the Central Committee of the Communist Party of China the General Office of the State Council. Opinions on Building a Higher-Level Public Service System for National Fitness. Available online at: http://www.gov.cn/zhengce/2022-03/23/content_5680908.htm (accessed March 23, 2022).

[8] World Health Organization. WHO Launches Portal for Global Data on the Health and Well-being of Older People. Available online at: https://www.who.int/news/item/01-10-2020-who-launches-portal-for-global-data-on-the-health-and-well-being-of-older-people (accessed October 1, 2020).

[9] Choi N. Relationship between health service use and health information technology use among older adults: analysis of the US National Health Interview Survey. J Med Int Res. (2011) 13:321–59. 10.2196/jmir.1753 21752784 PMC3221375

[10] Zhao JG Liu ZQ. The impact of Internet use on the health of the elderly. Chin J Popul Sci. (2020) 33:14–26+126. Available online at: https://kns.cnki.net/kcms/detail/detail.aspx?dbcode=CJFD&dbname=CJFDLAST2020&filename=ZKRK202005003&uniplatform=NZKPT&v=2atfsDAqZv38ZDQ1lAj6sCtv022TwP6QX3OwCa8YAek4EehHKX0JpdqPspWcEdNs35010361

[11] Zhang H Wang H Yan H Wang X. Impact of internet use on mental health among elderly individuals: a difference-in-differences study based on 2016-2018 CFPS Data. Int J Environ Res Public Health. (2021) 19:101. 10.3390/ijerph19010101 35010361 PMC8749999

[12] Sun K Zhou JJ. Understanding the impacts of Internet use on senior Citizens' social participation in China: evidence from longitudinal panel data. Telemat Informat. (2021) 59:101566. 10.1016/j.tele.2021.101566

[13] Heo J Chun S Lee S Lee KH Kim J. Internet use and well-being in older adults. Cyberpsychol Behav Soc Netw. (2015) 18:268–72. 10.1089/cyber.2014.0549 25919967

[14] Du P Wang B. How does internet use affect life satisfaction of the Chinese elderly? Popul Res. (2020) 44:3–17. Available online at: https://kns.cnki.net/kcms/detail/detail.aspx?dbcode=CJFD&dbname=CJFDLAST2020&filename=RKYZ202004001&uniplatform=NZKPT&v=5iSLgmb6sLgamyy0d1zU6eFOcXl4_CchzLen4Qy4DddnhryxRBrtbb3Pm7Ol1vo6

[15] Liu J Guo C. The Impact of Mobile Internet Application (APP) use on the physical and mental health of the elderly——Focusing on WeChat, WeChat moments and mobile payments. Popul Dev. (2021) 27:117–28. Available online at: https://kns.cnki.net/kcms/detail/detail.aspx?dbcode=CJFD&dbname=CJFDLAST2021&filename=SCRK202106010&uniplatform=NZKPT&v=VhvlJUnb5EeIFeQQDgwQhEU0F38mdo0UFex9b1frtvvp1NgDDYjcg-zg0j0QckgN

[16] Meischke H Eisenberg M Rowe S Cagle A. Do older adults use the internet for information on heart attacks? Results from A Survey of Seniors in King County, Washington. Heart Lung. (2005) 34:3–12. 10.1016/j.hrtlng.2004.06.006 15647729

[17] Jiang QL Chen ZH. Active aging among older surfers: a study on the mechanism of Internet use enhancing subjective well-being in the elderly. Modern Commun. (2021) 43:41–8. 10.19997/j.cnki.xdcb.2021.12.007

[18] Lv MY Peng XZ Lu MH. The effect of internet use on employment of the elderly. Econ Perspect. (2020) 60:77–97. Available online at: https://kns.cnki.net/kcms/detail/detail.aspx?dbcode=CJFD&dbname=CJFDLAST2021&filename=JJXD202010006&uniplatform=NZKPT&v=5G7A4FqQ6byr_WD4AzbWhLPrHcVgwaGe2EhExoqn4dD9QdLvpEqmyXmIxOJ3etZG

[19] Gilleard C Hyde M Higgs P. Community and communication in the third age: the impact of internet and cell phone use on attachment to place in later life in England. J Gerontol Ser B Psychol Sci Soc Sci. (2007) 62:S276–83. 10.1093/geronb/62.4.S276 17673541

[20] He H Yan CY. Segregation or integration: a study of the impact of internet use on community participation of Chinese Elderly. Popul J. (2022) 44:72–84. 10.16405/j.cnki.1004-129X.2022.02.006

[21] Li LQ Ding HF. Internet use, leisure time and physical exercise of rural residents—Empirical analysis based on 2018 CFPS data. Lanzhou Acad J. (2022) 42:108–22. Available online at: https://kns.cnki.net/kcms/detail/62.1015.c.20220126.1412.006.html

[22] Wu XH. Internet use and its influence on the Elderly: A study based on CSS (2013) questionnaire data analysis. J Yunnan Minzu Univ. (2017) 34:63–72. 10.13727/j.cnki.53-1191/c.2017.04.010

[23] Wang SQ Guo KL Lv WG. Will internet use promote physical exercise for the elderly in China? An empirical analysis based on CGSS. J Sports Res. (2021) 35:62–70. 10.15877/j.cnki.nsic.20210811.001

[24] Kearns A Whitley E. Associations of internet access with social integration, wellbeing and physical activity among adults in deprived communities: Evidence from a household survey. BMC Public Health. (2019) 19:1–15. 10.1186/s12889-019-7199-x 31266470 PMC6604194

[25] National Bureau of Statistics. IV. Statistical System and Classification Standards (17). Available online at: http://www.stats.gov.cn/tjzs/cjwtjd/201308/t20130829_74318.html (accessed February 21, 2022).

[26] Yang HL Wu YY Lin XY Xie L Zhang S Zhang SQ. Internet use, life satisfaction, and subjective well-being among the elderly: evidence from 2017 China General Social Survey. Front Public Health. (2021) 9:677643. 10.3389/fpubh.2021.677643 34268289 PMC8275954

[27] Murphy AP Duffield R Reid M. Tennis for physical health: acute age- and gender-based physiological responses to cardio tennis. J Strength Condit Res. (2014) 28:3172–8. 10.1519/JSC.0000000000000511 24796984

[28] Song L Li SZ. Intergenerational transition and its influence on health of the elderly in rural China: a study of gender differences across generations. J Chin Womens Stud. (2006) 14: 14–20+46. Available online at: https://kns.cnki.net/kcms/detail/detail.aspx?dbcode=CJFD&dbname=CJFD2006&filename=FNYJ200604002&uniplatform=NZKPT&v=fwnKJe6Quim3D1pURzKF_Th6oTmVj0F89oxB5Ntpmf0qySQKtKyHytkgMNhHh5bB

[29] Jia CC He WW. How does the children's taking care affect the elderly health?———Based on Propensity Score Matching (PSM) counterfactual estimate. Ningxia Soc Sci. (2020) 38:125–35. Available online at: https://kns.cnki.net/kcms/detail/detail.aspx?dbcode=CJFD&dbname=CJFDLAST2020&filename=LXSK202006017&uniplatform=NZKPT&v=OjfYvFXYnbtVv4Ewd83RjdK6sRqFHWXi5QbUCcXLcKs8CULAu0Ufv62n9YHxoi_Z

[30] Jiang XQ Wei M Zhang WJ. Study on the health status and influencing factors of china's aging population. Popul J. (2015) 37:46–56. Available online at: https://kns.cnki.net/kcms/detail/detail.aspx?dbcode=CJFD&dbname=CJFDLAST2015&filename=RKXK201502006&uniplatform=NZKPT&v=YV2AGn6AOrDSKHr2xteMwoLHw_eYuHrTF1aykIDuJGuDflmQ-UsDZrdZdD70ZuWr

[31] Shan N. The effect of cognitive aging on elderly women's willingness to care for the elderly. Chin J Gerontol. (2019) 39:2810–2. Available online at: https://kns.cnki.net/kcms/detail/detail.aspx?dbcode=CJFD&dbname=CJFDLAST2019&filename=ZLXZ201911085&uniplatform=NZKPT&v=j9Wok-VzFZ1GdlwnirtITgCBbTWK3p-kAXNsnXzvRCWUzki6BVSqUs39oc7wrV32

[32] Zhou SJ Yang HL Zhang JY. China's new poverty alleviation strategy for post-2020——Achievements, major goals, overall thinking and policy recommendations. Chin Public Administr. (2019) 34:6–11. 10.19735/j.issn.1006-0863.2019.11.01

[33] Cotten SR Ford G Ford S Hale TM. Internet use and depression among retired older adults in the United States. A longitudinal analysis. J Gerontol Ser B Psychol Sci Soci Sci. (2014) 69:763–71. 10.1093/geronb/gbu018 24671896

[34] Wang XH Shen KJ. On accurate assessment and influencing factors of healthy aging of the Chinese elderly: an empirical exploration of WHO's latest theoretical framework in China. J Yunnan Minzu Univ. (2021) 38:78–89. 10.13727/j.cnki.53-1191/c.20210903.013

[35] Afshin A Babalola D Mclean M Yu Z Ma W Chen CY. Information technology and lifestyle: a systematic evaluation of internet and mobile interventions for improving diet, physical activity, obesity, tobacco, and alcohol use. J Am Heart Assoc. (2016) 5:e003058. 10.1161/JAHA.115.003058 27581172 PMC5079005

[36] Yang L Song LJ. Decomposition of difference in healthy life expectancy among the elderly population in China. Popul Econ. (2022) 42:90–105. Available online at: https://kns.cnki.net/kcms/detail/11.1115.f.20211210.1847.002.html35926549

[37] Himmelmeier RM Nouchi R Saito T Burin D Wiltfang J. Kawashima R. Study protocol: does an acute intervention of high-intensity physical exercise followed by a brain training video game have immediate effects on brain activity of older people during stroop task in fMRI?-A randomized controlled trial with crossover design. Front Aging Neurosci. (2019) 11:260. 10.3389/fnagi.2019.00260 31619984 PMC6759467

[38] Han CL Xie ZZ Qu DX Liu HX Zhang ZW Luo WH. Multilevel model analysis of health factors in the elderly. Chin J Gerontol. (2021) 41:4574–7. Available online at: https://kns.cnki.net/kcms/detail/detail.aspx?dbcode=CJFD&dbname=CJFDLAST2021&filename=ZLXZ202120066&uniplatform=NZKPT&v=icp8mBBZcr5-FyhrgYMnsNOvGdfxT0XcxJMNx-hNcItPi3tFb5XXJ4CU2cpuVb7K

